# Cognitive Performance in Late Adolescence and the Subsequent Risk of Subdural Hematoma: An Observational Study of a Prospective Nationwide Cohort

**DOI:** 10.1371/journal.pmed.1001151

**Published:** 2011-12-27

**Authors:** Anna Nordström, Peter Nordström

**Affiliations:** 1 Rehabilitation Medicine, Department of Community Medicine and Rehabilitation, Umeå University, Umeå, Sweden; 2 Geriatric Medicine, Department of Community Medicine and Rehabilitation, Umeå University, Umeå, Sweden; University of Cambridge, United Kingdom

## Abstract

**Background:**

There are few identified risk factors for traumatic brain injuries such as subdural hematoma (SDH). The aim of the present study was to investigate whether low cognitive performance in young adulthood is associated with SDH later in life. A second aim was to investigate whether this risk factor was associated with education and physical fitness.

**Methods and Findings:**

Word recollection, logical, visuospatial, and technical performances were tested at a mean age of 18.5 years in a prospective nation-wide cohort of 440,742 men. An estimate of global intelligence was calculated from these four tests. Associations between cognitive performance, education, physical fitness, and SDH during follow-up were explored using Cox regression analyses. During a median follow-up of 35 years, 863 SDHs were diagnosed in the cohort. Low global intelligence was associated with an increased risk of SDH during follow-up (hazard ratio [HR]: 1.33, per standard deviation decrease, 95% CI = 1.25–1.43). Similar results were obtained for the other measures of cognitive performance (HR: 1.24–1.33, *p*<0.001 for all). In contrast, a high education (HR: 0.27, comparing more than 2 years of high school and 8 years of elementary school, 95% CI = 0.19–0.39), and a high level of physical fitness (HR: 0.76, per standard deviation increase, 95% CI = 0.70–0.83), was associated with a decreased risk of suffering from a SDH.

**Conclusions:**

The present findings suggest that reduced cognitive function in young adulthood is strongly associated with an increased risk of SDH later in life. In contrast, a higher level of education and a higher physical fitness were associated with a decreased risk of SDH.

*Please see later in the article for the Editors' Summary*

## Abstract 2

Anna and Peter Nordström analyzed a prospective nationwide cohort of 440,742 Swedish men and found that reduced cognitive function in young adulthood was associated with increased risk of subdural hematoma later in life, whereas a higher level of education and physical fitness were associated with a decreased risk.

## Editors' Summary

**Background:**

Every year, about 10 million people worldwide sustain a traumatic brain injury that needs medical attention or that proves fatal. Such injuries occur when the head is suddenly hit or jolted or when an object such as a bullet pierces the skull and enters the brain. Motor vehicle accidents are responsible for many traumatic brain injuries, but falls, assaults, and military action can also cause these serious injuries. The symptoms of a traumatic brain injury, which may not appear until many days after the injury, include loss of consciousness, headaches, dizziness, and nausea. Affected individuals can experience changes in their memory, concentration, or ability to think clearly (“cognitive” changes) and can have behavioral or emotional problems. Although the initial brain damage caused by trauma cannot be reversed, immediate medical treatment is essential to prevent further injury occurring. In particular, patients need to be monitored for “subdural hematoma,” a common outcome of traumatic brain injury in which blood from ruptured vessels collects between the brain and the skull. Subdural hematoma puts pressure on the brain and has to be removed surgically to prevent further brain damage.

**Why Was This Study Done?:**

Not everyone who has a traumatic brain injury develops subdural hematoma. If the factors that increase a person's risk of developing subdural hematoma could be identified, it might be possible to devise public-health interventions that would reduce the incidence of subdural hematomas. In this prospective population-based analysis, the researchers investigate whether low cognitive performance in early adulthood is associated with subdural hematoma later in life. Impaired cognitive functioning is sometimes recorded as a symptom of subdural hematoma but the researchers hypothesize that these cognitive deficits might have been present before the traumatic head injury that led to subdural hematoma. Low cognitive performance is associated with a reduced ability to compare objects and patterns (perceptual speed) and with impaired judgment, planning, and risk behavior (executive functions), so low cognitive performance might increase a person's risk of having an accident that results in a head injury and subdural hematoma.

**What Did the Researchers Do and Find?:**

The researchers calculated a global intelligence score for 440,742 male Swedish military conscripts (average age 18.5 years) from cognitive tests completed by the men between 1969 and 1978. They obtained information about diagnoses of subdural hematoma up to 40 years later among these men from medical records, and then used several statistical approaches to look for associations between cognitive performance, education (recorded during conscription assignment), physical fitness (measured during conscription assignment), and subsequent subdural hematoma. During the follow-up period, 863 subdural hematomas were diagnosed among the men. Conscripts with a low global intelligence score in early adulthood were more likely to develop subdural hematoma during later life than those with a high score. Specifically, when the men were divided into five groups (quintiles) on the basis of their global intelligence score, men with a score in the lowest quintile were more than twice as likely to develop subdural hematoma as those with a score in the highest quintile. By contrast, men who had had more than 2 years high school education were much less likely to develop subdural hematoma than those who had only had 8 years of elementary school education. A high level of physical fitness in early adulthood also reduced the risk of subdural hematoma.

**What Do These Findings Mean?:**

These findings suggest that low cognitive function in early adulthood is associated with subdural hematoma later in life, whereas high levels of education and physical fitness is associated with a decreased risk of subdural hematoma. Because this study was observational, these findings do not prove that low cognitive performance, low education level, or low physical fitness is causally linked to subdural hematoma. Other unidentified factors (confounders) shared by people with these characteristics might actually be responsible for the observed association between these factors and subdural hematoma. For example, poorly educated people might work in more hazardous environments than those who attended high school. However, if these findings can to be confirmed in other large studies, an exploration of the mechanistic basis of the associations reported here might eventually inform the development of public-health interventions designed to reduce the occurrence of subdural hematoma.

**Additional Information:**

Please access these Web sites via the online version of this summary at http://dx.doi.org/10.1371/journal.pmed.1001151.

- The US National Institute of Neurological Disorders and Stroke provides detailed information about traumatic brain injury (in English and Spanish)
- The US Centers for Disease Control and Prevention also provides detailed information about traumatic brain injury
- The UK National Health Service Choices website has an article about severe head injury that includes a personal story about a head injury sustained in a motor vehicle accident, and an article about subdural hematoma
- MedlinePlus provide links to further resources on traumatic brain injury and information on subdural hematoma; it also provides an interactive tutorial on traumatic brain injury (available in English and Spanish)
- The UK charity Headway, which works to improve life after brain injury, has a collection of personal stories about brain injury

## Introduction

The worldwide incidence of traumatic brain injury requiring medical attention or resulting in death was estimated at over 9.5 million in 1990 [1]. Subdural hematoma (SDH) is defined as a collection of blood between the brain and the dura mater usually from a laceration of bridge cortical veins associated with head injuries. SDH is the most common severe outcome of traumatic brain injury [2].

SDH can be divided into acute SDH or chronic SDH, which is diagnosed approximately 21 d after onset of bleeding [3],[4]. In a population-based study of traumatic brain injury in northern Sweden the proportion of isolated SDH in patients referred to a neurosurgery department was 51.2%, and of these 50.6% were diagnosed as chronic [2]. Symptoms of SDH include various aspects of impaired cognitive functioning. However, there have been very few studies that have actually investigated cognitive performance [5]–[7]. Cognitive impairment associated with SDH has most commonly been described as abnormal behavior or cognitive disturbances or deficits without any objective measurements [8]–[11]. The possibility of a latent development of dementia following head trauma has been suggested; the evidence for this, however, is inconclusive [12]. Another possibility is that low cognitive ability increases the risk of SDH and that the cognitive deficits seen after the occurrence of trauma were actually present before trauma. Low cognitive performance is associated with reduced perceptual speed [13] and impaired executive functions including poor judgment, planning, and risk behavior [14]–[16]. Thus, low cognitive functioning may be associated with several risk factors for accidents resulting in head injuries and SDHs [17],[18].

In the present study we hypothesized that low cognitive functioning in young adulthood may be associated with the later risk of a SDH. A second aim was to investigate whether this risk is associated with education and physical fitness.

## Methods

### Study Population

The cohort considered for inclusion in the present study consisted of all Swedish males that conscripted for compulsory military service between 1969 and 1978 (*n* = 457,302). These men represented approximately 97% of the male Swedish population born between 1951 and 1960 and the cohort was compiled from the Swedish Military Service Conscription Register (SMSCR). The background of the SMSCR has been described in detail previously [19]. Until recently, exemptions were granted only for incarcerated male subjects and those with documented severe chronic medical conditions or handicaps. For the present study men younger than 17 y at baseline or men, severely under or over weight (<40 or >170 kg), or of extreme stature (<140 or >215 cm), were excluded from further analysis (*n* = 9,545). We also excluded 6,975 additional men because of death or emigration before January, 1987 leaving 440,742 men for inclusion in the present study.

### Ethics Statement

The present study was approved by the local ethics committee of Umeå University and by the National Board of Health and Welfare in Sweden.

### Baseline Examination

All participants took part in a 2-d standardized intelligence and physical examination before conscription assignment to the Swedish Armed Forces at six regional conscription centers in Sweden. All conscripts underwent a medical examination by a physician and any disorders were diagnosed according to the Swedish version of International Classification of Disease (ICD), 8th edition. Weight, height, hearing, and vision were measured via standardized methods. Information on educational background was available for those who conscripted between 1972 and 1978 (*n* = 315,509), and was classified into four groups; 8 y of elementary school, 9 y of elementary school, 2 y of high school, and 3 y of high school or more. Physical capacity was assessed using an electrically braked bicycle ergometry test. In short, all participants with a normal resting electrocardiogram proceeded with a 5-min submaximal bicycling session at a work rate between 75 and 175 Watt (W) depending on body weight. Workload was then increased by 25 W/min until exhaustion occurred [20]. During the test, subjects were instructed to maintain pedal cadence between 60 and 70 revolutions per minute. Final work rate (physical fitness in W) was recorded. Data from the physical capacity test were available for 317,577 men who conscripted from 1972–1978.

### Cognitive Tests

The following tests of cognitive performance have been described previously [21],[22]. The verbal test of synonyms measures the ability to correctly choose the synonym of a given word from four alternatives. The visuospatial geometric perception test assesses the capability to identify the correct three dimensional object form a series of two-dimensional drawings. The logical/inductive performance test measures the capacity to understand written instructions and apply them to a problem solving task. The theoretical/technical test assesses a component of general knowledge through a mathematical/physics problem. The maximum score for each of the first three tests was 40 points while the maximum score for the mathematical/physics problem was 52 points. The administered tests started with simple questions that gradually became more difficult. A measure of global intelligence was estimated from the four tests. Each test was of equal importance and weighted on the basis of the mean score and variation of each test. The result was expressed as a score with minimum of zero and a maximum of 40 points.

### Diagnosis of SDH

Information on diagnosis of SDH between January, 1987 and December, 2009 was obtained through records from the National Hospital Discharge Register, covering all public inpatient care in Sweden, administered by the Center for Epidemiology at the National Board of Health and Welfare in Sweden. Diagnoses were recorded using the ICD version nine (1987–1997) and version ten (1998–2009). Deaths in the cohort occurring during the study period were collected through the National Cause of Death Register, administered by the Center for Epidemiology at the National Board of Health and Welfare in Sweden and emigration information was collected through the Statistics Sweden database.

### Statistics

Data are presented as mean ± standard deviation (SD) if not otherwise stated. Student's *t* tests for independent samples or Fischer's exact tests were performed to determine differences between subjects with and without SDH during follow-up. Associations between the explanatory variables at baseline and the later risk of SDH were tested using Cox proportional hazard models. Since age at baseline, differences over time as well as between the six test centers could introduce bias, age, year, and place of conscription were considered as confounders in all models. The independent effects of cognitive performance, education, and physical fitness were tested by including these variables in the same regression model. In this model all factors that were associated with the risk of a SDH according to Table 1 were considered as confounders. The joint effects of global intelligence and education for three groups of global intelligence and the four different groups of education were also assessed using Cox regression. The study endpoint for all Cox regression models was the date of an SDH, date of death, date of emigration, or December 31, 2009, whichever came first. The proportional hazard assumption was checked graphically using Kaplan-Meier curves. SPSS software for PCs (version 18.0; SPSS Inc.) was used for statistical analyses. All statistical tests were two-tailed. A *p*-value of less than 0.05 was considered statistically significant.

**Table 1:** Baseline characteristics of 440,742 men studied based on occurrence of SDH during follow-up.

Characteristics	SDH during Follow-up		*p*-Value
	Yes (*n* = 863)	No (*n* = 439,879)	
Age (y)	18.7±0.8	18.5±0.7	<0.001
Weight (kg)	66.8±9.7	68.1±9.7	<0.001
Height (cm)	177±7	179±7	<0.001
Physical fitness (W)	232±33	243±37	<0.001
**Cognitive performance**			
Global intelligence (0–40)	19.2±5.0	20.7±4.8	<0.001
Logical performance (1–40)	23.2±5.8	24.8±5.5	<0.001
Word recollection (1–40)	21.5±6.3	23.2±6.0	<0.001
Visuospatial performance (1–40)	13.2±4.1	14.1±3.9	<0.001
Technical performance (1–52)	30.6±8.2	32.3±8.0	<0.001
**Education**			
8 y of elementary school	20.6%	12.4%	
9 y of elementary school	36.0%	28.7%	
2 y of high school	35.4%	41.7%	
>2 y of high school	7.9%	17.2%	<0.001*
**Diagnoses**			
Impaired vision	0.9%	1.2%	0.41
Impaired hearing	12.3%	9.9%	0.03
Alcohol dependency	2.5%	0.5%	<0.001
Drug dependency	2.7%	0.6%	<0.001
Personality disorder	5.1%	1.9%	<0.001
Neurosis	7.3%	4.3%	0.001

*For comparing the distribution of the four different levels of education.

## Results

The present study followed 440,742 subjects from 18 y of age. During a median follow-up of 35 y (range 8–40), 863 subjects were diagnosed with a traumatic SDH at a median age of 46 y (range 28–59). Characteristics at baseline according to status during follow-up are shown in Table 1. Men diagnosed with a traumatic SDH were older, weighed less, and were shorter than those without SDH. In addition, men with a SDH diagnosis were less physically fit (*p*<0.001) and were more often diagnosed with substance abuse, personality disorders, and neurosis (*p*<0.01 for all measures). Men with SDH scored lower on all measures of cognitive performance at baseline (*p*<0.001 for all measures) and had a lower education (*p*<0.001) than the rest of the cohort. Using partial correlations and adjusting for age, year, and place of conscription, all measures of cognitive performance were associated with education (*r* = 0.24–0.42, *p*<0.001 for all correlations) and physical fitness (*r* = 0.12–0.19, *p*<0.001 for all correlations).

The most common cause of SDH was a fall in the same plane (Table 2). In total, falls explained about 58% of the SDHs occurring during follow-up. Other underlying causes included trauma during transportation and assault.

**Table 2:** Underlying mechanism for having a SDH during follow-up in a total of 440,742 men followed for a median of 35 y.

Underlying Mechanism	Global Intelligence		Logistic Performance		Word Recollection		Visuospatial Performance		Technical Performance		Education		Physical Fitness	
	HR	95% CI	HR	95% CI	HR	95% CI	HR	95% CI	HR	95% CI	HR	95% CI	HR	95% CI
Fall, same level (*n* = 207)	1.20	1.04–1.37	1.21	1.06–1.39	1.14	1.00–1.31	1.16	1.01–1.33	1.22	1.06–1.40	0.26	0.12–0.55	0.78	0.65–0.93
Fall, different level (*n* = 104)	1.32	1.09–1.60	1.30	1.07–1.58	1.19	0.98–1.45	1.25	1.03–1.52	1.24	1.02–1.50	0.26	0.10–0.73	0.75	0.59–0.94
Falls, unspecified (*n* = 190)	1.44	1.25–1.66	1.40	1.21–1.61	1.47	1.28–1.70	1.30	1.13–1.51	1.26	1.09–1.45	0.22	0.10–0.52	0.70	0.58–0.84
Trauma, motor (*n* = 111)	1.21	1.00–1.46	1.26	1.04–1.52	1.20	1.00–1.45	1.12	0.93–1.45	1.11	0.92–1.34	0.32	0.12–0.82	0.68	0.54–0.86
Transport, nonmotor (*n* = 76)	1.00	0.79–1.25	1.02	0.81–1.28	0.96	0.77–1.21	1.01	0.80–1.27	0.98	0.78–1.23	0.78	0.18–3.34	1.07	0.81–1.42
Assault (*n* = 67)	1.76	1.38–2.24	1.76	1.39–2.24	1.80	1.41–2.30	1.46	1.14–1.86	1.42	1.12–1.81	0.10	0.02–0.46	0.69	0.52–0.91
Other causes (*n* = 108)	1.65	1.37–1.99	1.56	1.29–1.88	1.47	1.22–1.77	1.54	1.27–1.86	1.47	1.22–1.77	0.47	0.16–1.34	0.82	0.65–1.03
**Total (** ***n*** ** = 863)**	1.33	1.25–1.43	1.33	1.24–1.42	1.29	1.20–1.38	1.25	1.17–1.33	1.24	1.16–1.32	0.27	0.19–0.39	0.76	0.70–0.83

HR are presented per SD decrease in cognitive performance according to underlying mechanism and in total. HRs for education are presented for those with more than 11 y of school with those with less than 9 y of school as reference, and per SD increase for physical fitness. The models were adjusted for age, conscription year, and place.

Using Cox regression (Model 1), after adjusting for age, year, and place of conscription, lower global cognitive performance (hazard ratio [HR]: 1.33 per SD decrease, 95% CI = 1.25–1.43 was associated with increased risk of SDH (Table 2). Similar results were found for the other measures of cognitive performance (Table 2). In contrast, a high education (HR: 0.27, comparing more than 2 y of high school and 8 y of elementary school 95% CI = 0.19–0.39), and a high level of physical fitness (HR: 0.76, per SD increase, 95% CI = 0.70–0.83) decreased the risk of SDH (Table 2). Adjusting the models including education or physical fitness also for global intelligence decreased the association slightly for education (HR: 0.40, 95% CI = 0.27–0.60) and for physical fitness (HR 0.81, per SD increase, 95% CI = 0.74–0.88). In the final regression model, global cognitive performance, education, physical fitness, all variables found to be associated with the risk of SDH according to Table 1, and also year and place of conscription, were used to explain the risk of SDH. In this model, global intelligence (HR 1.17, per SD decrease, 95% CI = 1.07–1.29), high education (HR: 0.46, 95% CI = 0.31–0.69, comparing >2 y of high school and 8 y of elementary school), and physical fitness (HR: 0.88, per SD increase, 95% CI = 0.79–0.97) were associated with SDH during follow-up.

Using Cox regression analysis, the independent effects of cognitive performance and education were further analyzed for low (≤15 points), medium (>15 points to ≤24 points), and high (>24 points) global intelligence (Table 3). Participants who were included in both the group with the highest education and the group with the highest global intelligence at baseline had 83% lower risk for SDH (HR: 0.17, 95% CI = 0.09–0.32) during follow-up, compared to those in the lowest group of both global intelligence and education.

**Table 3:** The independent effects of global intelligence and education at 18 y of age with respect to the risk of SDH during a median follow-up of 35 y in 440,742 men.

Global Intelligence	Education							
	8 y of Elementary School		9 y of Elementary School		2 y of High School		>2 y of High School	
	HR	95% CI	HR	95% CI	HR	95% CI	HR	95% CI
Low (<16 points)	1.00	—	1.03	0.58–1.85	0.71	0.41–1.23	0.76	0.17–3.45
Medium (>15–24 points)	0.78	0.51–1.18	0.54	0.35–0.82	0.37	0.25–0.56	0.14	0.07–0.30
High (>24 points)	0.53	0.23–1.21	0.45	0.25–0.82	0.35	0.22–0.57	0.17	0.09–0.32

Hazard ratios (HR) and 95% confidence intervals (95% CI) are presented for low, medium and high global intelligence and four different levels of education. The Cox regression models were adjusted for age at baseline, conscription place and year of conscription.

The associations between cognitive performance, education, physical fitness, and the risk of SDH were also analyzed for each underlying cause of SDH. Overall, the strongest associations were found when the underlying cause of SDH was assault, while an underlying cause of trauma during transportation not involving motor vehicles was not significantly associated with the risk of SDH for any of the tests of cognitive performance, education, or physical fitness (Table 2).

**Figure 1:**
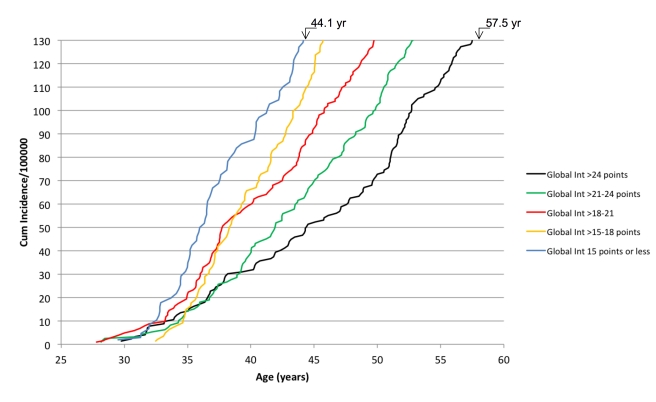
The cumulative incidence of SDH according to five different groups of global intelligence (Global Int). The follow-up was terminated at an incidence of 130 SDH per 100,000 subjects.

The associations between cognitive performance and the risk of SDH were further analyzed for quintiles of each measure, with the highest quintile as reference (Table 4). When comparing the highest to lowest quintiles of cognitive performance, global intelligence (HR: 2.31, 95% CI = 1.86–2.88), logical performance (HR: 2.14, 95% CI = 1.71–2.67), word recollection (HR: 1.89, 95% CI = 1.53–2.35), visuospatial performance (HR: 1.97, 95% CI = 1.58–2.47), and technical performance (HR: 1.85, 95% CI = 1.49–2.30) were found to be significantly associated with the risk of SDH after adjustment for age, place, and year of conscription. The cumulative incidence of SDH for five different groups of global intelligence is shown in Figure 1. Men in the lowest group of global intelligence (≤15 points) reached a cumulative incidence of 130/100,000 subjects with an SDH diagnosis 13 y earlier than those with a global intelligence of ≥25 points. Assuming a linear relationship, the cumulative incidence increased by 4.8 SDHs/year (95% CI = 4.6–4.9) in the group with highest cognitive performance compared to 9.8 SDHs/year (95% CI = 9.5–10.1) in the group with lowest cognitive performance.

**Table 4:** The risk of SDH for quintiles of cognitive performance.

Cognitive Performance	Quintile 1	Quintile 2		Quintile 3		Quintile 4		Quintile 5	
	HR	HR	95% CI	HR	95% CI	HR	95% CI	HR	95% CI
**Global intelligence**	1.00	1.18	0.92–1.51	1.24	0.97–1.58	1.69	1.35–2.13	2.31	1.86–2.88
**Logical performance**	1.00	0.95	0.71–2.67	1.34	1.06–1.69	1.56	1.23–1.98	2.14	1.71–2.67
**Word recollection**	1.00	0.93	0.73–1.18	1.23	0.97–1.55	1.47	1.16–1.85	1.89	1.53–2.35
**Visuospatial performance**	1.00	1.03	0.81–1.32	1.17	0.92–1.47	1.27	1.01–1.60	1.97	1.58–2.47
**Technical performance**	1.00	1.28	1.01–1.62	1.36	1.07–1.72	1.57	1.25–1.97	1.85	1.49–2.30

The highest quintile of each measure of cognitive performance was used as reference. HRs and 95% CIs are presented. The Cox regressions models were adjusted for age at baseline, conscription place, and year of conscription.

## Discussion

The results of the present study indicate that low cognitive performance in young adulthood is associated with an increased risk of sustaining a traumatic SDH later in life. Moreover, general education and objective measures of physical fitness were associated both with cognitive performance in young adulthood and the risk of SDH later in life. In a final model, a high compared to a low education and physical fitness decreased the risk of a SDH by 54% and 12%, respectively. We suggest that such estimated effects may be important on a public health level if the results could be replicated in other epidemiological settings.

The clinical importance of cognitive performance as a risk factor for the future risk of SDH may be illustrated in Figure 1. The cumulative incidence of SDH was followed in five different groups with respect to global intelligence. In our population of men, 12% had an estimated global intelligence of at most 15 points, and 24% of the men had a score of at least 25 points. When comparing these groups, men in the group with the lower score reached a specified cumulative incidence of SDHs 13 y earlier. Thus, these results suggest that a high cognitive functioning is associated with a delayed occurrence of SDH by more than a decade.

SDHs have many negative consequences including pain, debility, and reduced self-dependency [23],[24], and therefore, manipulation of environmental factors that may affect cognitive performance could prove to be beneficial to reduce the risk of SDHs. In the present study, we especially evaluated the importance of education and objective measures of physical fitness. Education and physical fitness were strongly associated with all measures of cognitive performance at baseline. Education and physical fitness were also independently associated with the risk of SDH during follow-up. With respect to education, men with at least 3 y of high school had a 73% decreased risk of suffering SDH during follow-up, compared to those with 8 y of elementary school. Moreover, most of the estimated effects of education on the risk of SDH were independent of cognitive performance. In support of this result, higher education is associated with more desk jobs, which may be associated with fewer environmental hazards than manual labor. A high physical fitness at baseline was also associated with a decreased risk of SDH. Thus, every SD increase in physical fitness decreased the risk of SDH by 24% during follow-up. To better evaluate the importance of education and physical fitness, both these factors were included in the final regression model to explain the risk of SDH, adjusting for global cognitive performance and all significant confounders at baseline. At least 3 y of high school was then found to reduce the risk of SDH by 54%, and a higher physical fitness was found to be associated with a 12%. decreased risk. Confirmation of these findings in other large cohorts would be useful to validate these associations. An exploration of the mechanistic basis for these associations might allow the construction of public health interventions aimed at reducing the population incidence of SDH, define high risk groups in whom these interventions might be most usefully trialed, and identify intermediate endpoints that might provide further mechanistic explanation of benefit (if detected). For example, increasing the proportion of young males receiving secondary school education may result in reduced alcohol and/or substance abuse, less job-related environmental hazards, and could, by this effect, reduce the risk of head injury.

A few studies have investigated cognitive performance in relation to chronic SDH and these suggest that reduced cognitive function is present after acquired SDH [5]–[7]. Also occurrence of mild traumatic head injury, usually referred to as concussion, has been associated with lower cognitive performance [25],[26]. However, there are no previous data linking SDHs or concussions to premorbid cognitive performance. Our data suggest that the level of cognitive performance prior to SDH may in part be responsible for cognitive functioning after SDH acquisition. The results from the present study could also have implications for the rehabilitation process following SDH. A low premorbid cognitive performance could affect the outcome of SDH [27]. The predominantly metabolic pathophysiology during the initial period after a traumatic brain injury, may be more difficult for individuals with low cognitive capacity to overcome resulting in an impeded degree of recovery and adaptation [28],[29].

Given that the studied cohort consisted of only men inferences from these results to women should be made with caution. We cannot exclude that there are confounders not accounted for in the present study that could influence the associations found. Low socio-economic class would be one such factor that probably influences the education level of the participants in present study. Education at baseline was only available for the 315,509 men that conscripted from 1972–1978. In addition, the present study was observational and thus inferences about causality should also be made with great caution. However, some of the findings in the present study may suggest a cause–effect relationship. The associations found between cognition, education, physical fitness, and the risk of SDH were independent of medical conditions and other confounders accounted for at baseline. There was also a dose-dependent relationship between cognitive performance and the risk of SDH. Furthermore, the associations between the main independent variables (cognitive performance, education, and physical fitness) and the risk of SDH showed a consistent pattern for the different underlying causes of SDH. Thus, the strongest association for any measure of cognitive performance, education, physical fitness, and SDH was found when the underlying cause was assault. With respect to this result it is of interest to note that reduced cognitive performance is associated with problem behavior and impaired judgment [14],[15].

In summary, in a large population-based analysis, low cognitive performance at 18 y of age was strongly associated with traumatic SDH during a median of 35 y of follow-up. In contrast, a high education and physical fitness was associated with a decreased risk of traumatic SDH independent of the level of cognitive performance. In addition, the current results suggest that the lower cognitive functioning seen after traumatic brain injury may be influenced by premorbid cognitive performance.

## Abbreviations

HR: hazard ratio
SD: standard deviation
SDH: subdural hematoma
